# Methyl 5-meth­oxy-2-nitro-4-[3-(piperidin-1-yl)prop­oxy]benzoate

**DOI:** 10.1107/S1600536809005418

**Published:** 2009-02-21

**Authors:** Min Zhang, Ran-Zhe Lu, Lu-Na Han, Wen-Bin Wei, Hai-Bo Wang

**Affiliations:** a College of Science, Nanjing University of Technology, Xinmofan Road No. 5 Nanjing, Nanjing 210009, People’s Republic of China

## Abstract

In the mol­ecule of the title compound, C_17_H_24_N_2_O_6_, the dihedral angle between the four coplanar atoms of the piperidine ring and the benzene ring is 39.2 (1)°.

## Related literature

For general background, see: Knesl *et al.* (2006 ▶). For bond-length data, see: Allen *et al.* (1987 ▶).
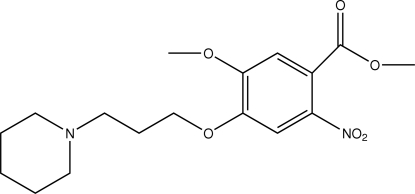


## Experimental

### 

#### Crystal data

C_17_H_24_N_2_O_6_
*M* *_r_* = 352.38
Monoclinic, 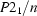

*a* = 10.073 (2) Å
*b* = 11.140 (2) Å
*c* = 16.161 (3) Å
β = 97.23 (3)°
*V* = 1799.1 (6) Å^3^
*Z* = 4
Mo *K*α radiation
μ = 0.10 mm^−1^
*T* = 293 K
0.30 × 0.20 × 0.20 mm

#### Data collection

Enraf–Nonius CAD-4 diffractometer
Absorption correction: ψ scan (North *et al.*, 1968 ▶) *T*_min_ = 0.971, *T*_max_ = 0.981
3458 measured reflections
3262 independent reflections
1950 reflections with *I* > 2σ(*I*)
*R*_int_ = 0.042
3 standard reflections every 200 reflections intensity decay: 1%

#### Refinement

*R*[*F* ^2^ > 2σ(*F* ^2^)] = 0.068
*wR*(*F* ^2^) = 0.175
*S* = 1.01
3262 reflections
226 parameters
H-atom parameters constrained
Δρ_max_ = 0.25 e Å^−3^
Δρ_min_ = −0.29 e Å^−3^

### 

Data collection: *CAD-4 EXPRESS* (Enraf–Nonius, 1994 ▶); cell refinement: *CAD-4 EXPRESS*; data reduction: *XCAD4* (Harms & Wocadlo, 1995 ▶); program(s) used to solve structure: *SHELXS97* (Sheldrick, 2008 ▶); program(s) used to refine structure: *SHELXL97* (Sheldrick, 2008 ▶); molecular graphics: *SHELXTL* (Sheldrick, 2008 ▶); software used to prepare material for publication: *PLATON* (Spek, 2009 ▶).

## Supplementary Material

Crystal structure: contains datablocks global, I. DOI: 10.1107/S1600536809005418/hb2910sup1.cif

Structure factors: contains datablocks I. DOI: 10.1107/S1600536809005418/hb2910Isup2.hkl

Additional supplementary materials:  crystallographic information; 3D view; checkCIF report

## Figures and Tables

**Fig. 1.**
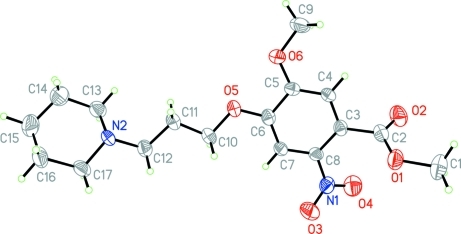
The molecular structure of (I) showing 50% displacement ellipsoids for the non-hydrogen atoms.

## supplementary crystallographic information

### Comment

As part of our ongoing studies on quinazoline derivatives (Knesl *et al.*, 2006), we report herein the crystal structure of the title compound, (I).

In the molecule of the title compound (Fig. 1), the bond lengths (Allen *et al.*,  1987) and angles are within normal ranges. Ring A (C4-C9) is, of course, planar.

### Experimental

A solution of methyl 4-(3-chloropropoxy)-5-methoxy-2-nitrobenzoate (0.013 mol), potassium carbonate (0.052mol), sodium iodide (0.026mol) in acetonitrile (33 mL) was stirred for 5-10 min at room temperature. Piperidine (0.040mol) was added and this mixture heated to reflux for 3 h. Reaction progress was monitored by TLC. Solid material was removed by filtration and washed with acetone. The combined filtrates were evaporated and the dark product obtained dissolved in dichloromethane (30 ml) and extracted with water (4 × 10 ml). The organic phase was dried (Na_2_SO_4_), decolorized (charcoal), filtered and evaporated to afford the product (yield; 71.2%,) as an amber oil.  Yellow blocks of (I) were obtained by slow evaporation of an methanol solution.

### Refinement

H atoms were positioned geometrically, with C-H = 0.93, 0.97 and 0.96 Å for aromatic, methylene and methyl H, respectively, and constrained to ride on their parent atoms, with U_iso_(H) = xU_eq_(C), where x = 1.5 for methyl H and x = 1.2 for all other H atoms.

### Crystal data

**Table:**

C_17_H_24_N_2_O_6_	*F*(000) = 752
*M**_r_* = 352.38	*D*_x_ = 1.301 Mg m^−^^3^
Monoclinic, *P*2_1_/*n*	Mo *K*α radiation, λ = 0.71073 Å
Hall symbol: -P 2yn	Cell parameters from 25 reflections
*a* = 10.073 (2) Å	θ = 10–13°
*b* = 11.140 (2) Å	µ = 0.10 mm^−^^1^
*c* = 16.161 (3) Å	*T* = 293 K
β = 97.23 (3)°	Block, yellow
*V* = 1799.1 (6) Å^3^	0.30 × 0.20 × 0.20 mm
*Z* = 4	

### Data collection

**Table:**

Enraf–Nonius CAD-4 diffractometer	1950 reflections with *I* > 2σ(*I*)
Radiation source: fine-focus sealed tube	*R*_int_ = 0.042
graphite	θ_max_ = 25.3°, θ_min_ = 2.2°
ω/2θ scans	*h* = 0→12
Absorption correction: ψ scan (North *et al.*, 1968)	*k* = 0→13
*T*_min_ = 0.971, *T*_max_ = 0.981	*l* = −19→19
3458 measured reflections	3 standard reflections every 200 reflections
3262 independent reflections	intensity decay: 1%

### Refinement

**Table:**

Refinement on *F*^2^	Primary atom site location: structure-invariant direct methods
Least-squares matrix: full	Secondary atom site location: difference Fourier map
*R*[*F*^2^ > 2σ(*F*^2^)] = 0.068	Hydrogen site location: inferred from neighbouring sites
*wR*(*F*^2^) = 0.175	H-atom parameters constrained
*S* = 1.01	*w* = 1/[σ^2^(*F*_o_^2^) + (0.06*P*)^2^ + 2.6*P*] where *P* = (*F*_o_^2^ + 2*F*_c_^2^)/3
3262 reflections	(Δ/σ)_max_ < 0.001
226 parameters	Δρ_max_ = 0.25 e Å^−^^3^
0 restraints	Δρ_min_ = −0.29 e Å^−^^3^

### Special details

**Table:**

Geometry. All esds (except the esd in the dihedral angle between two l.s. planes) are estimated using the full covariance matrix. The cell esds are taken into account individually in the estimation of esds in distances, angles and torsion angles; correlations between esds in cell parameters are only used when they are defined by crystal symmetry. An approximate (isotropic) treatment of cell esds is used for estimating esds involving l.s. planes.
Refinement. Refinement of F^2^ against ALL reflections. The weighted R-factor wR and goodness of fit S are based on F^2^, conventional R-factors R are based on F, with F set to zero for negative F^2^. The threshold expression of F^2^ > 2sigma(F^2^) is used only for calculating R-factors(gt) etc. and is not relevant to the choice of reflections for refinement. R-factors based on F^2^ are statistically about twice as large as those based on F, and R- factors based on ALL data will be even larger.

### Fractional atomic coordinates and isotropic or equivalent isotropic displacement parameters (Å^2^)

**Table:**

	*x*	*y*	*z*	*U*_iso_*/*U*_eq_	
O1	1.0523 (2)	0.3714 (2)	0.40317 (15)	0.0487 (7)	
O2	1.0082 (3)	0.1757 (2)	0.38640 (16)	0.0563 (7)	
O3	0.7178 (3)	0.5462 (3)	0.32890 (18)	0.0746 (10)	
O4	0.7681 (3)	0.3835 (3)	0.39767 (17)	0.0627 (8)	
O5	0.8037 (2)	0.4291 (2)	0.03763 (13)	0.0404 (6)	
O6	0.9697 (2)	0.2550 (2)	0.05972 (14)	0.0408 (6)	
N1	0.7693 (3)	0.4455 (3)	0.33404 (18)	0.0445 (8)	
N2	0.6436 (2)	0.7187 (2)	−0.17424 (16)	0.0314 (6)	
C1	1.1303 (4)	0.3499 (4)	0.4829 (2)	0.0633 (12)	
H1A	1.1646	0.4247	0.5061	0.095*	
H1B	1.2035	0.2973	0.4756	0.095*	
H1C	1.0748	0.3134	0.5199	0.095*	
C2	0.9964 (3)	0.2764 (3)	0.3627 (2)	0.0395 (8)	
C3	0.9287 (3)	0.3122 (3)	0.27838 (19)	0.0325 (7)	
C4	0.9752 (3)	0.2621 (3)	0.20979 (19)	0.0310 (7)	
H4A	1.0391	0.2015	0.2174	0.037*	
C5	0.9293 (3)	0.2995 (3)	0.13026 (19)	0.0303 (7)	
C6	0.8349 (3)	0.3941 (3)	0.1185 (2)	0.0328 (7)	
C7	0.7857 (3)	0.4424 (3)	0.18563 (19)	0.0316 (7)	
H7A	0.7229	0.5039	0.1782	0.038*	
C8	0.8291 (3)	0.4000 (3)	0.26530 (19)	0.0323 (7)	
C9	1.0556 (4)	0.1520 (3)	0.0662 (2)	0.0520 (10)	
H9A	1.0762	0.1303	0.0118	0.078*	
H9B	1.0112	0.0863	0.0896	0.078*	
H9C	1.1368	0.1706	0.1016	0.078*	
C10	0.7202 (3)	0.5334 (3)	0.0212 (2)	0.0368 (8)	
H10A	0.7556	0.5999	0.0560	0.044*	
H10B	0.6301	0.5165	0.0332	0.044*	
C11	0.7190 (3)	0.5643 (3)	−0.0687 (2)	0.0380 (8)	
H11A	0.6879	0.4957	−0.1027	0.046*	
H11B	0.8094	0.5827	−0.0796	0.046*	
C12	0.6288 (3)	0.6713 (3)	−0.0931 (2)	0.0404 (8)	
H12A	0.5364	0.6474	−0.0922	0.048*	
H12B	0.6488	0.7343	−0.0519	0.048*	
C13	0.5908 (4)	0.6370 (3)	−0.2416 (2)	0.0462 (9)	
H13A	0.4957	0.6258	−0.2401	0.055*	
H13B	0.6340	0.5595	−0.2326	0.055*	
C14	0.6130 (5)	0.6835 (4)	−0.3253 (2)	0.0601 (11)	
H14A	0.5713	0.6294	−0.3679	0.072*	
H14B	0.7083	0.6848	−0.3293	0.072*	
C15	0.5571 (4)	0.8070 (4)	−0.3413 (2)	0.0618 (11)	
H15A	0.4602	0.8042	−0.3471	0.074*	
H15B	0.5837	0.8382	−0.3928	0.074*	
C16	0.6085 (4)	0.8881 (3)	−0.2698 (2)	0.0509 (10)	
H16A	0.5661	0.9661	−0.2779	0.061*	
H16B	0.7041	0.8991	−0.2691	0.061*	
C17	0.5809 (4)	0.8370 (3)	−0.1868 (2)	0.0431 (9)	
H17A	0.6163	0.8905	−0.1421	0.052*	
H17B	0.4851	0.8299	−0.1860	0.052*	

### Atomic displacement parameters (Å^2^)

**Table:**

	*U*^11^	*U*^22^	*U*^33^	*U*^12^	*U*^13^	*U*^23^
O1	0.0500 (15)	0.0488 (15)	0.0426 (14)	−0.0022 (12)	−0.0119 (11)	0.0005 (12)
O2	0.0646 (18)	0.0432 (16)	0.0596 (17)	0.0030 (13)	0.0019 (13)	0.0194 (13)
O3	0.077 (2)	0.079 (2)	0.068 (2)	0.0443 (18)	0.0102 (16)	−0.0039 (17)
O4	0.0541 (17)	0.083 (2)	0.0536 (17)	0.0055 (15)	0.0154 (13)	0.0086 (16)
O5	0.0454 (14)	0.0389 (13)	0.0358 (13)	0.0092 (11)	0.0006 (10)	0.0073 (11)
O6	0.0398 (13)	0.0429 (14)	0.0400 (13)	0.0103 (11)	0.0065 (10)	−0.0039 (11)
N1	0.0279 (15)	0.064 (2)	0.0408 (17)	0.0085 (15)	0.0003 (12)	−0.0051 (16)
N2	0.0313 (14)	0.0257 (14)	0.0365 (14)	0.0058 (11)	0.0010 (11)	0.0049 (12)
C1	0.054 (3)	0.085 (3)	0.046 (2)	−0.005 (2)	−0.0129 (18)	0.001 (2)
C2	0.0359 (19)	0.042 (2)	0.0406 (19)	0.0052 (16)	0.0040 (15)	0.0079 (17)
C3	0.0281 (17)	0.0323 (18)	0.0358 (18)	−0.0028 (14)	−0.0015 (13)	0.0014 (14)
C4	0.0257 (16)	0.0305 (17)	0.0369 (18)	0.0034 (13)	0.0052 (13)	−0.0002 (14)
C5	0.0249 (16)	0.0276 (17)	0.0385 (18)	−0.0009 (13)	0.0038 (13)	−0.0059 (14)
C6	0.0273 (17)	0.0325 (18)	0.0369 (18)	−0.0028 (13)	−0.0024 (13)	0.0020 (14)
C7	0.0249 (16)	0.0341 (18)	0.0356 (17)	0.0060 (14)	0.0025 (13)	0.0005 (14)
C8	0.0280 (17)	0.0339 (18)	0.0348 (17)	0.0045 (14)	0.0039 (13)	−0.0040 (14)
C9	0.051 (2)	0.045 (2)	0.060 (2)	0.0147 (18)	0.0097 (18)	−0.0103 (19)
C10	0.0323 (18)	0.0338 (18)	0.044 (2)	0.0052 (14)	0.0025 (14)	0.0066 (15)
C11	0.0389 (19)	0.0320 (18)	0.0419 (19)	0.0012 (15)	0.0009 (15)	0.0072 (15)
C12	0.040 (2)	0.041 (2)	0.0394 (19)	0.0064 (16)	0.0013 (15)	0.0054 (16)
C13	0.051 (2)	0.041 (2)	0.043 (2)	0.0069 (17)	−0.0037 (16)	−0.0023 (17)
C14	0.077 (3)	0.058 (3)	0.043 (2)	0.007 (2)	−0.0016 (19)	−0.004 (2)
C15	0.070 (3)	0.071 (3)	0.043 (2)	0.011 (2)	−0.0005 (19)	0.013 (2)
C16	0.055 (2)	0.045 (2)	0.054 (2)	0.0093 (18)	0.0088 (18)	0.0187 (19)
C17	0.045 (2)	0.0331 (19)	0.051 (2)	0.0104 (16)	0.0081 (16)	0.0028 (17)

### Geometric parameters (Å, °)

**Table:**

O1—C2	1.332 (4)	C9—H9A	0.9600
O1—C1	1.442 (4)	C9—H9B	0.9600
O2—C2	1.187 (4)	C9—H9C	0.9600
O3—N1	1.235 (4)	C10—C11	1.492 (4)
O4—N1	1.240 (4)	C10—H10A	0.9700
O5—C6	1.362 (4)	C10—H10B	0.9700
O5—C10	1.439 (4)	C11—C12	1.521 (4)
O6—C5	1.352 (4)	C11—H11A	0.9700
O6—C9	1.432 (4)	C11—H11B	0.9700
N1—C8	1.422 (4)	C12—H12A	0.9700
N2—C12	1.439 (4)	C12—H12B	0.9700
N2—C17	1.464 (4)	C13—C14	1.492 (5)
N2—C13	1.466 (4)	C13—H13A	0.9700
C1—H1A	0.9600	C13—H13B	0.9700
C1—H1B	0.9600	C14—C15	1.498 (6)
C1—H1C	0.9600	C14—H14A	0.9700
C2—C3	1.499 (4)	C14—H14B	0.9700
C3—C4	1.375 (4)	C15—C16	1.506 (6)
C3—C8	1.398 (4)	C15—H15A	0.9700
C4—C5	1.375 (4)	C15—H15B	0.9700
C4—H4A	0.9300	C16—C17	1.514 (5)
C5—C6	1.416 (4)	C16—H16A	0.9700
C6—C7	1.360 (4)	C16—H16B	0.9700
C7—C8	1.389 (4)	C17—H17A	0.9700
C7—H7A	0.9300	C17—H17B	0.9700
			
C2—O1—C1	117.1 (3)	O5—C10—H10B	110.2
C6—O5—C10	117.9 (2)	C11—C10—H10B	110.2
C5—O6—C9	118.3 (3)	H10A—C10—H10B	108.5
O3—N1—O4	120.9 (3)	C10—C11—C12	111.3 (3)
O3—N1—C8	119.1 (3)	C10—C11—H11A	109.4
O4—N1—C8	119.9 (3)	C12—C11—H11A	109.4
C12—N2—C17	111.4 (3)	C10—C11—H11B	109.4
C12—N2—C13	112.3 (3)	C12—C11—H11B	109.4
C17—N2—C13	110.2 (3)	H11A—C11—H11B	108.0
O1—C1—H1A	109.5	N2—C12—C11	113.3 (3)
O1—C1—H1B	109.5	N2—C12—H12A	108.9
H1A—C1—H1B	109.5	C11—C12—H12A	108.9
O1—C1—H1C	109.5	N2—C12—H12B	108.9
H1A—C1—H1C	109.5	C11—C12—H12B	108.9
H1B—C1—H1C	109.5	H12A—C12—H12B	107.7
O2—C2—O1	125.0 (3)	N2—C13—C14	112.1 (3)
O2—C2—C3	124.1 (3)	N2—C13—H13A	109.2
O1—C2—C3	110.5 (3)	C14—C13—H13A	109.2
C4—C3—C8	118.2 (3)	N2—C13—H13B	109.2
C4—C3—C2	117.5 (3)	C14—C13—H13B	109.2
C8—C3—C2	124.0 (3)	H13A—C13—H13B	107.9
C5—C4—C3	121.5 (3)	C13—C14—C15	112.3 (3)
C5—C4—H4A	119.3	C13—C14—H14A	109.1
C3—C4—H4A	119.3	C15—C14—H14A	109.1
O6—C5—C4	125.2 (3)	C13—C14—H14B	109.1
O6—C5—C6	115.3 (3)	C15—C14—H14B	109.1
C4—C5—C6	119.5 (3)	H14A—C14—H14B	107.9
C7—C6—O5	126.1 (3)	C14—C15—C16	109.5 (3)
C7—C6—C5	119.6 (3)	C14—C15—H15A	109.8
O5—C6—C5	114.4 (3)	C16—C15—H15A	109.8
C6—C7—C8	120.1 (3)	C14—C15—H15B	109.8
C6—C7—H7A	120.0	C16—C15—H15B	109.8
C8—C7—H7A	120.0	H15A—C15—H15B	108.2
C7—C8—C3	120.9 (3)	C15—C16—C17	111.7 (3)
C7—C8—N1	119.2 (3)	C15—C16—H16A	109.3
C3—C8—N1	119.8 (3)	C17—C16—H16A	109.3
O6—C9—H9A	109.5	C15—C16—H16B	109.3
O6—C9—H9B	109.5	C17—C16—H16B	109.3
H9A—C9—H9B	109.5	H16A—C16—H16B	107.9
O6—C9—H9C	109.5	N2—C17—C16	109.7 (3)
H9A—C9—H9C	109.5	N2—C17—H17A	109.7
H9B—C9—H9C	109.5	C16—C17—H17A	109.7
O5—C10—C11	107.4 (3)	N2—C17—H17B	109.7
O5—C10—H10A	110.2	C16—C17—H17B	109.7
C11—C10—H10A	110.2	H17A—C17—H17B	108.2
			
C1—O1—C2—O2	−1.6 (5)	C4—C3—C8—C7	−5.0 (5)
C1—O1—C2—C3	−175.4 (3)	C2—C3—C8—C7	169.6 (3)
O2—C2—C3—C4	−55.8 (5)	C4—C3—C8—N1	173.9 (3)
O1—C2—C3—C4	118.1 (3)	C2—C3—C8—N1	−11.5 (5)
O2—C2—C3—C8	129.6 (4)	O3—N1—C8—C7	−25.2 (5)
O1—C2—C3—C8	−56.6 (4)	O4—N1—C8—C7	153.9 (3)
C8—C3—C4—C5	2.3 (5)	O3—N1—C8—C3	155.8 (3)
C2—C3—C4—C5	−172.7 (3)	O4—N1—C8—C3	−25.0 (5)
C9—O6—C5—C4	7.4 (4)	C6—O5—C10—C11	171.1 (3)
C9—O6—C5—C6	−174.4 (3)	O5—C10—C11—C12	177.9 (3)
C3—C4—C5—O6	−180.0 (3)	C17—N2—C12—C11	−166.2 (3)
C3—C4—C5—C6	1.8 (5)	C13—N2—C12—C11	69.6 (4)
C10—O5—C6—C7	5.7 (4)	C10—C11—C12—N2	168.9 (3)
C10—O5—C6—C5	−173.2 (3)	C12—N2—C13—C14	−176.9 (3)
O6—C5—C6—C7	178.3 (3)	C17—N2—C13—C14	58.3 (4)
C4—C5—C6—C7	−3.4 (4)	N2—C13—C14—C15	−55.0 (4)
O6—C5—C6—O5	−2.8 (4)	C13—C14—C15—C16	51.9 (5)
C4—C5—C6—O5	175.6 (3)	C14—C15—C16—C17	−54.0 (5)
O5—C6—C7—C8	−178.2 (3)	C12—N2—C17—C16	175.3 (3)
C5—C6—C7—C8	0.7 (5)	C13—N2—C17—C16	−59.3 (4)
C6—C7—C8—C3	3.5 (5)	C15—C16—C17—N2	58.5 (4)
C6—C7—C8—N1	−175.4 (3)		
